# PSPI: A deep learning approach for prokaryotic small protein identification

**DOI:** 10.3389/fgene.2024.1439423

**Published:** 2024-07-10

**Authors:** Matthew Weston, Haiyan Hu, Xiaoman Li

**Affiliations:** ^1^ Department of Computer Science, University of Central Florida, Orlando, FL, United States; ^2^ Burnett School of Biomedical Science, College of Medicine, University of Central Florida, Orlando, FL, United States

**Keywords:** small proteins, prokaryotes, deep learning, long short-term memory, machine learning

## Abstract

Small Proteins (SPs) are pivotal in various cellular functions such as immunity, defense, and communication. Despite their significance, identifying them is still in its infancy. Existing computational tools are tailored to specific eukaryotic species, leaving only a few options for SP identification in prokaryotes. In addition, these existing tools still have suboptimal performance in SP identification. To fill this gap, we introduce PSPI, a deep learning-based approach designed specifically for predicting prokaryotic SPs. We showed that PSPI had a high accuracy in predicting generalized sets of prokaryotic SPs and sets specific to the human metagenome. Compared with three existing tools, PSPI was faster and showed greater precision, sensitivity, and specificity not only for prokaryotic SPs but also for eukaryotic ones. We also observed that the incorporation of (*n*, *k*)-mers greatly enhances the performance of PSPI, suggesting that many SPs may contain short linear motifs. The PSPI tool, which is freely available at https://www.cs.ucf.edu/∼xiaoman/tools/PSPI/, will be useful for studying SPs as a tool for identifying prokaryotic SPs and it can be trained to identify other types of SPs as well.

## 1 Introduction

Small proteins (SPs), typically consisting of 100 amino acids (AA) or fewer, are indispensable components in cells, serving critical functions such as cell defense, adaptive immunity, and intercellular communication (Sberro et al., 2019). For instance, the SP MgrB regulates the activity of the sensor kinase PhoQ in response to antimicrobial peptides during bacterial infection (Jiang et al., 2023). Toddler, another SP, facilitates cell migration during embryonic gastrulation (Pauli et al., 2014). Because of the pivotal roles of SPs, identifying SPs is imperative for understanding cellular processes.

The identification of SPs is still in its infancy. Traditionally, open reading frames (ORFs) are at least 303 nucleotide long and proteins encoded by these ORFs are thus at least 100 AA long (Su et al., 2013). Although these cutoffs are somewhat arbitrary, they are necessary because the shorter cutoffs would have resulted in a much higher false positive prediction of genes and proteins. Because of such a historical constraint, despite their widespread existence, SPs have only started to be appreciated and studied in the last decade or so.

Experimentally, SPs are often identified by mass spectrometry or ribosome profiling (Kaltashov et al., 2013; Brar and Weissman, 2015; Ahrens et al., 2022). These experimental methods are originally designed for regular proteins of at least 100 AA long, while later adapted for SP identification. They have enabled our rudimentary understanding of SPs. Note that these experiments can only uncover SPs under a given experimental condition, as the activity of SPs or small ORFs (sORFs) coding them is condition-specific. Because it is impossible to do experiments under every condition, it is imperative to develop computational approaches for systematically predict SPs directly from nucleotide or peptide sequences without additional experimental data input.

A handful of computational methods have been developed for predicting SPs without additional experimental data (Miravet-Verde et al., 2019; Zhu and Gribskov, 2019; Durrant and Bhatt, 2021; Yu et al., 2021; Zhang et al., 2021; Zhang et al., 2022). Most of these methods are created to target SPs or sORFs in eukaryotes, such as csORF-Finder, MiPepid, and DeepCPP.

csORF-Finder is a tool focused on coding sORFs and can identify sORFs in the coding sequence and non-coding regions of DNA. It showed better performance than other existing methods (Zhang et al., 2022). MiPepid applies a logistic regression model with nucleotide tetramer features to predict whether a sequence contains sORFs coding for SPs (Zhu and Gribskov, 2019). DeepCPP is a deep-learning tool for RNA coding potential prediction, including sORFs coding SPs (Zhang et al., 2021).

There are also three computational methods for prokaryotic SP identification directly from genomic sequences: RanSEPs (Miravet-Verde et al., 2019), SmORFinder (Durrant and Bhatt, 2021), and PsORF (Yu et al., 2021). RanSEPs and SmORFinder predict SPs in the input prokaryotic genome or metagenome. They thus require prior knowledge of certain genome features, such as a fraction of known ORFs and the genome structure. Such a prerequisite prevents their wide application to unassembled prokaryotic sequences or short sequences. Although PsORF considers short sequences as input for SP identification, it is no longer accessible. Therefore, there is a great need to develop computational methods for prokaryotic SP identification.

To fill this gap, we present in this study a long short-term memory (LSTM) based approach for prokaryotic SP identification (PSPI). PSPI uses the AA sequences, codified as a series of binary vectors, and a parameter we’ve called (n, k)-mers, which is a form of gap k-mer, to identify SPs. Through testing on known prokaryotic SPs, human metagenome prokaryotic SPs, and known eukaryotic SPs, as well as their randomly permuted negatives and known non-coding negatives, we demonstrated that PSPI reliably distinguishes known SPs from random or known negatives. Compared with three existing approaches, PSPI significantly outperforms in nearly every instance in regard to precision, sensitivity, specificity, F1 score, AUROC, and AUPR. While PSPI is developed to identify prokaryotic SPs, we discovered it has additional capabilities for identifying eukaryotic SPs as well. Specifically, it does not have the same high false positive rate that other tools have, and it performs better than them if trained using eukaryotic data instead. Additionally, we explored the crucial features for accurate SP prediction and identified gapped dimers as particularly significant. In the following, we detail the PSPI method, its evaluation and comparison with other methods, and the pivotal features enhancing its accuracy.

## 2 Materials and Methods

### 2.1 Positive data

We collected prokaryotic SPs from three sources. First, we extracted data from the prokaryotic dataset Pro-6318 by Yu et al. (2021). This dataset comprises 6,318 sORFs from 56 prokaryotic species, with average and median lengths of 76 and 78 AA, respectively. Secondly, we retrieved SPs from the UniprotKB database (UniProt, 2023). We filtered for bacterial SPs with length ≤100 AA (taxonomy_id:2) and removed any SPs already present in Pro-6318, resulting in 24,433 SP sequences with an average length of 75 AA and a median length of 79 AA. This SP collection was designated as UniprotKB-pro. Thirdly, we collected SPs from the study by Sberro et al. (2019). They analyzed 1773 human body site metagenomes and computationally predicted 4,539 clusters of short peptide sequences and their corresponding nucleotide sequences. Each cluster comprises sequences from at least eight assembled contigs (“species”), indicating sequence conservation across species and thus likely representing authentic SPs. After filtering out sequences containing unknown AA, those with missing nucleotides in homologs, containing intermittent stop codons, or already in the Pro-6318 dataset we retained 27,794 potential SPs and their corresponding nucleotide sequences, termed microbiome_hs.

We also collected eukaryotic SPs from UniprotKB, similar to the prokaryotic SPs from UniprotKB described above. The distinction is the use of eukaryote taxonomy ID 2759 instead of taxonomy ID 2. This yielded 22,075 SPs, averaging 57 AA in length with a median length of 62 AA. We called this set UniprotKB-euk. The UniprotKB-euk set serves to explore the differences between prokaryotic and eukaryotic SPs and to assess the efficacy of PSPI in predicting eukaryotic SPs.

### 2.2 Negative data

We also constructed negative data in two ways. One was to permute the SP sequences. Given a SP sequence, we converted each of its AA into one of the codons that corresponds to it, followed by appending a stop codon to the end of the converted sequence. If an AA had multiple codons that correspond to it, one of those codons would be randomly chosen. We then randomly shuffled the obtained nucleotide sequence while preserving the start and stop codons. Finally, we converted the resulting nucleotide sequence back into a peptide sequence. Notably, we avoided permuting the original SP sequence to generate a negative sequence, as the permuted sequence shares the same AA composition, potentially still being a SP sequence. If we already had the sORF sequence, we directly permuted it accordingly. If a stop codon occurred in the middle of the permuted sequence, it was randomly substituted with a non-stop codon. This yielded four sets of negatives, corresponding to three positive sets of prokaryotic SPs and one positive set of eukaryotic SPs collected above.

The other way we constructed negatives was using eukaryotic microRNAs. A large number of microRNAs exist, and the short microRNAs are unlikely to contain sORFs. We could also include other non-coding RNAs, however obtaining many other non-coding sequences that were unlikely to contain SPs was challenging. We downloaded the hairpin.fa file from miRbase (Kozomara et al., 2019), which contains the ∼70 nucleotide long precursor microRNA sequences. We concatenated all sequences into a single sequence and then randomly partitioned it into non-overlapping substrings, each ranging from 30 to 300 nucleotides in length. Any stop codons within these substrings were randomly replaced with non-stop codons. We then converted each nucleotide sequence into its corresponding protein sequence, yielding 69,153 negative sequences from microRNAs. This set of negatives was then randomly divided into microRNA subsets 1, 2, 3, and four which contained 17,289, 17,290, 17,288, and 17,286 negatives, respectively.

### 2.3 Training and testing data

**FIGURE 1:**
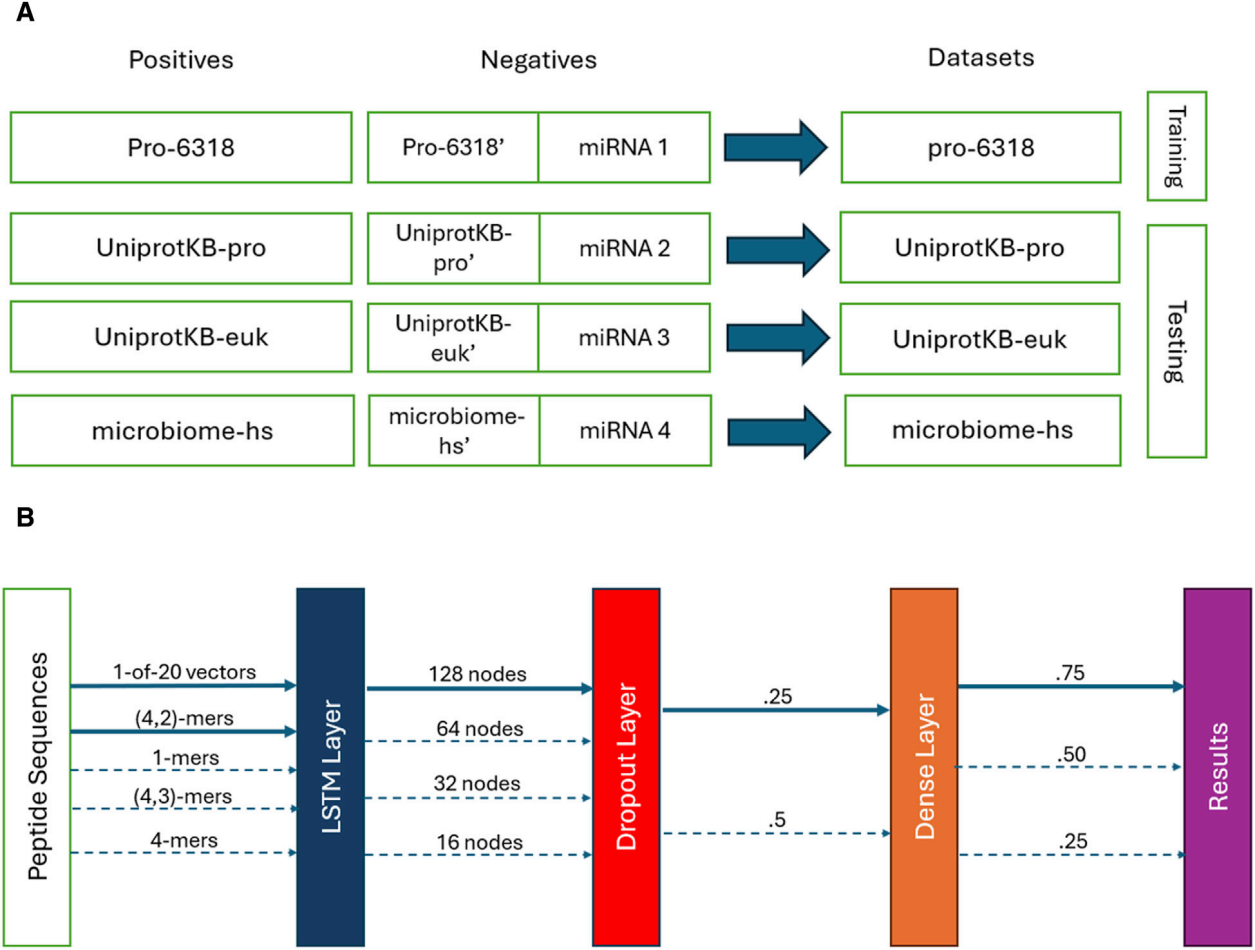
**(A)** Training and testing datasets. **(B)** The PSPI model architectures. Solid lines show the final parameters used. Dotted ones are other parameters evaluated.

We used the SPs obtained from the Pro-6318 dataset as the positive training data and paired them with the permuted SPs generated from them alongside the microRNA subset 1 as the training negatives. This combination of the training positives and negatives, called the pro-6318 training dataset below (Figure 1A), was employed to train the PSPI model. We tested the trained PSPI on three independent testing datasets: the UniprotKB-pro testing dataset, the microbiome-hs testing dataset, and the UniprotKB-euk testing dataset. Similar to the training dataset, each testing dataset comprised of one of the three remaining sets of positive SPs (UniprotKB-pro, microbiome-hs, UniprotKB-euk) as positives, juxtaposed with the corresponding permuted SPs and one of the remaining microRA subsets as negatives (Figure 1A). For instance, in the UniprotKB-pro testing data, its positives were the SPs in UniprotKB-pro, and its negatives were the permuted SPs from UniprotKB-pro alongside microRNA subset 2.

### 2.4 The PSPI model and its input

We developed a deep learning model called PSPI to predict whether an input peptide sequence is an SP (Figure 1B). PSPI adopts a LSTM-based architecture. LSTMs are a type of recurrent neural networks, specializing in learning order dependence within data which includes short, long, and variable length patterns in sequences (Hochreiter and Schmidhuber, 1997; Talukder et al., 2021; Athaya et al., 2023). LSTMs have been used to identify different types of proteins in the past (Yi et al., 2019; Youmans et al., 2020; Qin et al., 2023). Given the significance of AA order in protein folding and interaction, we employed LSTM to model the ordered AA within an SP.

The PSPI model architecture, implemented using the Keras Python Package (Chollet, 2021), constitutes a multi-layer sequential model. The initial layer is an LSTM layer, which converts the input data into a 128-dimensional vector. Next, a dropout layer with a dropout rate of .25 is applied, followed by a dense layer and a Sigmoid activation layer, yielding a single decimal score within the range [0,1] (Figure 1B). We classified all sequences with a score ≥ .75 as positive and those below as negative. We assessed different version of the LSTM model which output a 16, 32, 64, or 128-dimensional vector and settled on 128 as it gave us the best overall results. Similarly, we assessed a dropout rate of .25 and .5 and settled on .25.

We coded the sequences in two different ways to train different PSPI models. One way was to code each sequence as a binary vector of 2000 dimensions, in which each AA corresponds to a vector of 20 dimensions, with only one of its entries having a value of one and the rest being zeros. For sequences shorter than 100 AA, the positions after their maximal lengths are represented by 20-dimensional zero vectors. That is, short sequences are paddled with 20-dimensional zero vectors to reach the maximal length of 100 AA.

The other way we coded a sequence was by using the aforementioned 2000 binary numbers together with the count of (*n, k*)-mers. An (*n, k*)-mer is a gap k-mer in peptide substrings which is at most *n* AA long. For instance, ACD, AC.D, and A.C.D are the same (7, 3)-mer, while A….C.D is not a (7, 3)-mer (longer than 7). With this said, (*n, k*)-mers are different from the gapped k-mers mentioned in previous studies (Zhang et al., 2021), where every gapped k-mer has a fixed length. The (*n, k*)-mers considered here mimic short linear motifs in proteins (Van Roey et al., 2014), whose functions are determined by their ordered *k* AA and do not depend on their tertiary structures. Note that when *k* > 2, the number of possible (*n, k*)-mers is too large to train PSPI well. We thus used degenerated AA. That is, we considered AA with similar chemical and physical properties as one type and grouped the 20 AA into the following nine groups (Yi et al., 2019): [AGILPV], [FW], [M], [C], [ST], [Y], [D], [HKR], and [NQ]. We also tried other possible groupings and found that PSPI performed slightly better with the above grouping. For each sequence in the training dataset, in addition to the 2000 binary numbers describing its AA in order, a vector of 9^k^ is added to represent the count of the 9^k^ (*n, k*)-mers in this sequence when k > 2. For k ≤ 2, a vector of $20^{k}$ is used, since we use regular AA rather than the degenerated groups. We input such vectors 2000+9^k^ (k > 2) or 2000 + 20^k^ (k ≤ 2) for the training sequences to train the PSPI model. Because of the limited training data, we consider *k* from two to 4. Because protein linear motifs are 3–10 AA long, we considered different *n* from 3 to 10.

### 2.5 Comparison with other methods

We compared PSPI with three representative tools, csORF-Finder, MiPepid, and DeepCPP, on the testing datasets (Zhu and Gribskov, 2019; Zhang et al., 2021; Zhang et al., 2022). We selected these tools for comparison because they are specifically designed to predict SPs from sequences. Moreover, csORF-Finder demonstrated superior performance in their own recent evaluation; MiPepid performed well in the study of csORF-Finder; and DeepCPP is a deep learning-based approach and expected to perform well. Because these tools use the nucleotide sequences as inputs, we generated the corresponding nucleotide sequences of the testing peptide sequences in our testing datasets when running the tools.

With csORF-Finder, we configured it to predict SPs using its H.sapiens-CDS model and ran the following command for each testing dataset stored in separated files: “python3 csorf_finder_predict_sORFs.py -i <filename> -o <filename>.csv -m H. sapiens-CDS”. CDS refers to the coding sequence regions of mRNA. csORF-finder has models trained using both CDS and nonCDS regions. In their validation testing, CDS models consistently performed better than the non-CDS models, hence we opted for the CDS model for comparison (Zhang et al., 2022).

With MiPepid, we ran the following command for each of our testing datasets: “python3./src/mipepid.py <filename> <filename>.csv”. MiPepid attempts to find sORFs in a sequence without the requirement to set any specific species. It can thus predict an input sequence in any eukaryotic species as an sORF or its substrings as a sORF. The MiPepid results we reported refer to all sequences instead of their substrings it considers a potential sORF, since each sequence in our testing datasets was either a sORF or not a sORF.

DeepCPP includes a file DeepCPP.ipynb used to run the tool. For each testing dataset, we gave the.ipynb file the command “test_model(’./input_files/’, ‘./output_files/’, ‘<filename>’, ‘human’, ‘sorf’)”. Similarly, we configured DeepCPP to predict SPs using its human sORF model.

### 2.6 Performance measures

We used precision, sensitivity, specificity, F1, AUROC, and AUPR as performance measurements when comparing PSPI to other models and when we determined the ideal training data. We used only the AUROC and AUPR to determine how implementing different (*n, k*)-mers impacted the model.

## 3 Results

### 3.1 PSPI predicted prokaryotic SPs with high performance

We trained the original PSPI model on the pro-6318 training dataset with the 2000-dimensional binary vector representation of an input sequence (Material and Methods). We evaluated this PSPI model on three independent testing datasets (Table 1). PSPI had a high performance in predicting prokaryotic SPs. It had an area under the receiver operating characteristic curve (AUROC) of 0.994 and an area under the precision-recall curve (AUPR) of 0.986 on the UniprotKB-pro testing dataset. The AUROC and AUPR were similar but slightly lower on the microbiome-hs testing dataset, indicating that the UniprotKB annotated SPs are of higher quality than the computationally inferred SPs in microbiome-hs. The AUROC and AUPR were at least 19% lower on the UniprotKB-euk testing dataset, suggesting that the eukaryotic SPs may have different characteristics from their prokaryotic counterparts.

**TABLE 1:** The performance of PSPI on three testing datasets.

PSPI	Dataset	Precision	Sensitivity	Specificity	F1	AUROC	AUPR
Original PSPI	UniprotKB-pro	0.936	0.935	0.978	0.936	0.989	0.979
	UniprotKB-euk	0.876	0.416	0.955	0.564	0.762	0.770
	microbiome-hs	0.832	0.922	0.906	0.875	0.970	0.956
PSPI from eukaryotic data	UniprotKB-pro	0.917	0.793	0.965	0.850	0.961	0.939
	UniprotKB-euk	0.868	0.867	0.933	0.868	0.954	0.942
	microbiome-hs	0.843	0.934	0.921	0.839	0.947	0.923
Final PSPI model	UniprotKB-pro	0.959	0.947	0.986	0.953	0.994	0.988
	UniprotKB-euk	0.931	0.478	0.973	0.631	0.852	0.849
	microbiome-hs	0.891	0.938	0.942	0.914	0.982	0.972

To assess the impact of the positive training dataset on PSPI performance, we trained additional PSPI models using three subsets of SPs from UniprotKB-pro. We randomly divided the 31,125 SPs in UniprotKB-pro into three non-overlapping similar-sized subsets. Each subset served as positive training data, while their corresponding permuted SPs and the microRNA negatives from the original PSPI model were retained as negatives to train a different PSPI model. Testing these models on independent datasets revealed AUROC and AUPR values very close to the original ones (e.g., AUROC 0.985 *versus* 0.994 on the UniprotKB-pro testing data), indicating minimal influence of the positive SPs on model performance. The similar AUROC and AUPR also suggests that SPs in pro-6318 are as reliable as those in UniprotKB-pro.

Subsequently, we investigated how the choice of the training negatives impacted PSPI accuracy.

Two PSPI models were trained with SPs from pro-6318 as positives and employed either permuted SPs from pro-6318 or one set of microRNA negatives as negatives, instead of the combined set used in the original model. When we tested these models on the same dataset, the model showed near constant performance at identifying the positives but varied greatly with the negatives when the training and testing sources differed. For example, specificity drastically differed when using permuted SPs as negatives during training and microRNA negatives during testing, and *vice versa*. This discrepancy in specificity suggests distinct characteristics between permuted and microRNA negatives. Hence, utilizing combined negatives in the original PSPI model yielded improved performance. Comparing results in Tables 1, 2 shows employing both negative data sources in training enhanced the model’s ability to correctly label negative data (specificity: 0.978) without compromising its capacity to label positive data (sensitivity: 0.935).

**TABLE 2:** Average scores when the model is trained using only one type of negative data.

Training negatives	Testing negatives	Precision	Sensitivity	Specificity	F1	AUROC	AUPR
Permutation	Permutation	0.965	0.952	0.942	0.959	0.987	0.992
Permutation	microRNA	0.677	0.952	0.728	0.792	0.937	0.905
microRNA	microRNA	0.976	0.976	0.986	0.976	0.996	0.994
microRNA	Permutation	0.783	0.976	0.551	0.869	0.929	0.959

### 3.2 PSPI had superior performance to three existing tools

**FIGURE 2:**
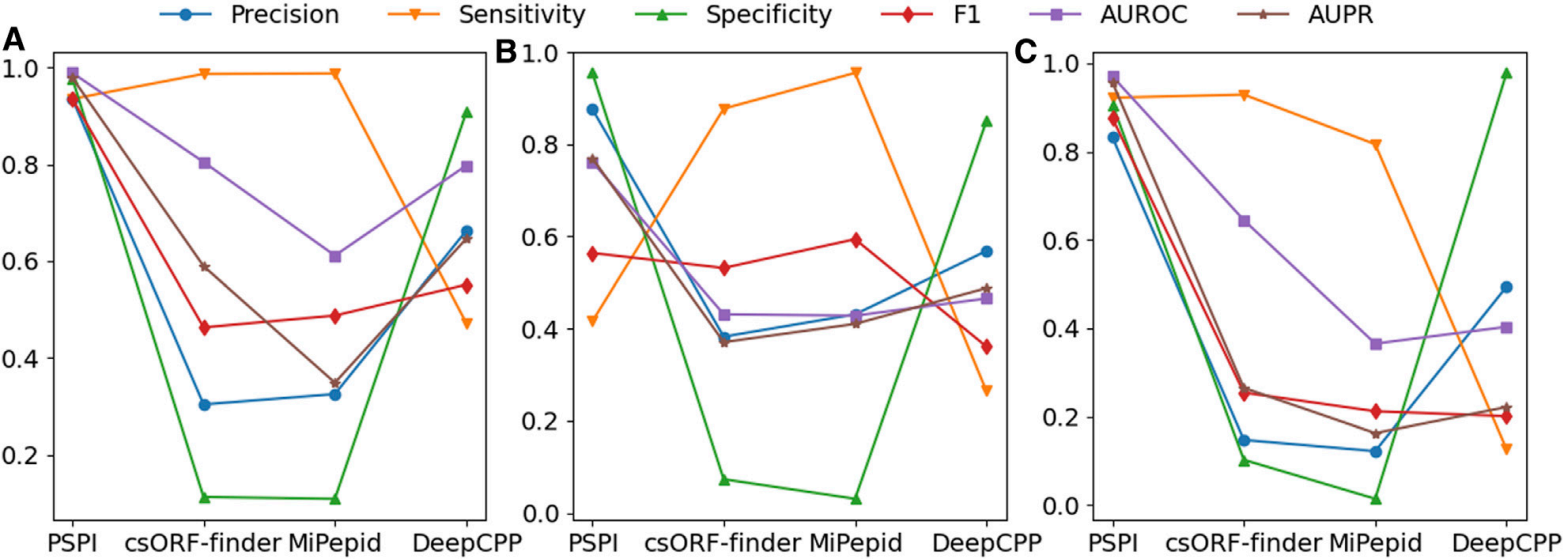
The Comparison of PSPI, csORF-finder, MiPepid, and DeepCPP on three testing datasets. **(A)** UniprotKB-pro; **(B)** UniprotKB-euk; and **(C)** microbiome-hs.

We evaluated the original PSPI model with csORF-Finder, MiPepid, and DeepCPP on the three independent testing datasets (Figure 2). These comparing tools were all used for eukaryotic SP identification. We chose them because they are specifically designed for SP identification. Moreover, the existing few tools for prokaryotic SP identification cannot be applied to the short testing sequences we had or are inaccessible.

PSPI had superior performance to these tools in almost every metric we compared (Figure 2). For instance, when tested on the UniprotKB-pro testing dataset, PSPI had a precision of 0.936, a sensitivity or recall of 0.935, a specificity of 0.978, an AUROC of 0.989, and an AUPR of 0.979, while the three existing tools had the best precision of 0.663 (DeepCPP), the best sensitivity of 0.988 (MiPepid), the best specificity of 0.908 (DeepCPP), the best AUROC of 0.805 (csORF-Finder), and the best AUPR of 0.646 (DeepCPP). Since the three tools were designed for eukaryotic SP identification, it would be fair to compare them on the UniprotKB-euk testing dataset. Again, PSPI consistently performed much better than the three tools in every metric except the sensitivity and F1 scores. Because PSPI had a better AUPR and AUROC on the UniprotKB-euk testing dataset, it could have better sensitivity, specificity, and F1 score than other tools when using different cutoffs instead of the default one for prokaryotic SPs.

As pointed out above, PSPI did not perform as well on eukaryotic SPs as on prokaryotic SPs (Table 1). This was likely because PSPI was trained on the prokaryotic SPs. To see whether PSPI would perform better on eukaryotic SPs if it was trained on them, we trained another PSPI model using the UniprotKB-euk dataset. We set aside one-third of all positives and negatives for training the new PSPI model and set the remaining two-thirds for testing. We found that the performance of the new PSPI model significantly improved on the eukaryotic SPs (Table 3), with much better performance than the three tools in every metric except the sensitivity. The sensitivity for eukaryotic sequences (0.867) became comparable to the other three tools, losing only to MiPepid (0.955).While its performance on prokaryotic SPs was not as good as the original PSPI model on prokaryotic SPs, it has comparable AUPR and AUROC, suggesting that the eukaryotic SPs have certain unique unknown features different from the prokaryotic SPs.

**TABLE 3:** AUROC and AUPR of the PSPI models with various (*n*, *k*)-mers.

Dataset		Baseline	1mers	Dimers	Trimers	Tetramer	All
AUROC	UniprotKB-pro	0.989	0.989	0.994	0.991	0.990	0.993
	UniprotKB-euk	0.762	0.765	0.852	0.823	0.814	0.80
	microbiome-hs	0.970	0.978	0.982	0.976	0.969	0.979
AUPR	UniprotKB-pro	0.979	0.978	0.988	0.985	0.982	0.988
	UniprotKB-euk	0.769	0.771	0.849	0.820	0.814	0.816
	microbiome-hs	0.956	0.953	0.972	0.963	0.955	0.970

We also compared the runtime of the original PSPI model, csORF-Finder, MiPepid, and DeepCPP on two datasets with 3,500 and 6,500 sequences, respectively. We did not include the time it took to build the PSPI model from scratch when we measured the running time of PSPI. All tests were done on an Acer x86_64 laptop using an Intel^®^ Core™ i3-8130U 2.2 GHz processor with four cores. The laptop was equipped with 16 GB of random access memory. PSPI took roughly 450–500 s to build the model. However, it took only 9.30 and 16.02 s to process 3,500 and 6,500 sequences, respectively. This is better than all other tools since the best of the three tools, MiPepid, took 18.93 s and 39 s, respectively. Through additional testing, we also noticed that the running time of PSPI increases linearly with respect to the number of input sequences.

### 3.3 Gapped (*n, k*)-mers enhanced the performance of PSPI

Previous studies have highlighted the significance of gapped motifs in SP predictions (Zhang et al., 2021). It is also suggested that many SPs may not have tertiary structures (Neidigh et al., 2002; Kubatova et al., 2020). We thus hypothesize that SPs are likely to contain short linear motifs such as the (*n, k*)-mers (Van Roey et al., 2014). Short linear motifs often exist in unstructured protein regions and are usually responsible for signaling. The actual AA sequence rather than the structure determines the function of these motifs.

We investigated how different gapped (*n, k*)-mers would affect the performance of PSPI. Recall that the original PSPI was trained on the pro-6318 training dataset, with each input sequence represented by a binary vector of 2000 dimensions. To utilize gapped (*n, k*)-mers, we trained PSPI on the same pro-6318 training dataset, with each input sequence represented by a vector of 2000+9^k^ (k > 2) or 2000 + 20^k^ (k ≤ 2) dimensions (Material and Methods).

We studied how the AUROC and AUPR of the trained PSPI model changed with different (*n*, *k*)-mers when it was tested on the UniprotKB-pro and microbiome-hs datasets. We considered *n* in [3,10], the typical range of short linear motifs. We only considered *k = 2* to 4, because of the limited number of SPs in the training dataset. The AUROC and AUPR had their largest or close-to-the-largest values for different *k* when *n = 4*. For instance, on the UnitprotKB-pro testing dataset, when *k = 2*, the PSPI model using (*4*, *2*)-mers would give us the second largest AUROC (0.9967) and AUPR (0.9972), which was only barely less than the largest AUROC (0.9968) and AUPR (0.9973) when *n = 5*. When *k = 3*, the PSPI model using (*4*, *3*)-mers would have the largest AUROC (0.9959) and AUPR (0.9965). We thus fixed *n = 4*.

We then studied how the AUROC and AUPR of the trained PSPI model changed with different (*4*, *k*)-mers when tested on all three testing datasets. Our baseline model used only a 2000-dimension binary vector representation of an input sequence. We compared the baseline model with the PSPI models trained with the addition of 1-mers (the frequency of 20 AA), (*4*, 2)-mers (dimers), (*4*, *3*)-mers (trimers), (*4*, *4*)-mers (tetramer), or all of them together (Table 3). We observed that improvements in correctly identifying SPs on the UniprotKB-pro testing dataset were minimal. However, there were noticeable improvements in identifying the microbiome-hs dataset and great improvements in the UniprotKB-euk dataset. The different degrees of improvement on different testing datasets are likely due to the different improvement space on these datasets, with much more space to improve on the UniprotKB-euk testing dataset. This analysis also implied that there are subtle signals like (*n*, *k*)-mers in SPs. In all cases, the model trained with (*4*, 2)-mers always performed best (Tables 1, 3).

Due to these findings, we decided our final version of PSPI would use a 2000-dimension binary vector and a 400-dimension count vector to represent the AA sequence and (*4, 2*)-mer count respectively.

## 4 Discussion

We developed PSPI, a tool utilizing LSTM to predict SPs in prokaryotes. We demonstrated its superior performance over existing tools in both accuracy and speed, particularly in identifying prokaryotic SPs. We also showed that with proper training on eukaryotic SPs, PSPI can effectively predict SPs in eukaryotes.

Incorporating the (*n*, *k*)-mer feature to represent input sequences improves the model performance. (*n*, *k*)-mers are modified k-mers, which allow a flexible number of gaps inside them. They help to represent the relative order of AA without the exponential growth burden of the parameters that would happen with regular k-mers. In our study, we found that the incorporation of (*4, 2*)-mers improved the PSPI performance most. (*4, 2*)-mers may represent undiscovered signals in SPs, which warrant further investigation.

Notably, the distinction between identifying coding sORFs and SPs influenced tool performance.

All tools we compared are intended to identify coding sORFs whereas PSPI is meant to identify SPs. Because of this difference, other tools all did better than themselves when the negatives were microRNAs than when the negatives were permuted SPs. Certain parameters these tools used, such as 3-mer or 4-mer counts, may not be nearly as capable of distinguishing coding from non-coding sORFs when the number of nucleotides in a sequence is multiples of three. It also explains why these tools had high accuracy in their original testing on sORFs while not having even close accuracy here on the SP sequences.

Interestingly, we observed that the trained PSPI model using eukaryotic SPs was still capable of identifying prokaryotic SPs (Table 1). The eukaryote-trained model had a noticeably low sensitivity score when identifying sequences in UniprotKB-pro (0.793), but it still maintained a high AUROC and AUPR (0.961 and 0.939), which implied that it was the high threshold score rather than the model itself that caused it to be unable to identify prokaryotic SPs. This may also indicate common traits between prokaryotic and eukaryotic SPs albeit with differences.

In the future, several directions may be explored to improve the accuracy of SP identification further. First, one may want to have better negative datasets to predict SPs. Our research showed that the negatives greatly affect the prediction accuracy. More representative negatives obtained in the future may produce better models. Second, we should systematically identify short linear motifs in SPs. Our research suggested that short linear motifs may exist in SPs. However, the identification of these short linear motifs is still challenging. Existing tools are often designed for a specific genome, not a mixture of genomes. Moreover, their accuracy is insufficient to prevent the high false positive rate in predictions. Finally, one may study the difference between eukaryotic and prokaryotic SPs. Our study implied the difference between them, but had no clue what exactly the difference is. Addressing these problems may lead to more accurate prediction of SPs and a better understanding of their functions.

## Data Availability

The original contributions presented in the study are included in the article/Supplementary Material, further inquiries can be directed to the corresponding authors.
